# Integrative Clustering in Mass Spectrometry Imaging for Enhanced Patient Stratification

**DOI:** 10.1002/prca.201800137

**Published:** 2019-01-04

**Authors:** Benjamin Balluff, Achim Buck, Marta Martin‐Lorenzo, Frédéric Dewez, Rupert Langer, Liam A. McDonnell, Axel Walch, Ron M.A. Heeren

**Affiliations:** ^1^ Maastricht MultiModal Molecular Imaging institute (M4I) Maastricht University 6229 ER Maastricht The Netherlands; ^2^ Research Unit Analytical Pathology Helmholtz Zentrum München 85764 Oberschleißheim Germany; ^3^ Institute of Pathology University of Bern CH‐3008 Bern Switzerland; ^4^ Fondazione Pisana per la Scienza ONLUS 56017 Pisa Italy

**Keywords:** cancer, integrative clustering, mass spectrometry imaging, prognosis

## Abstract

**Scope:**

In biomedical research, mass spectrometry imaging (MSI) can obtain spatially‐resolved molecular information from tissue sections. Especially matrix‐assisted laser desorption/ionization (MALDI) MSI offers, depending on the type of matrix, the detection of a broad variety of molecules ranging from metabolites to proteins, thereby facilitating the collection of multilevel molecular data.

Lately, integrative clustering techniques have been developed that make use of the complementary information of multilevel molecular data in order to better stratify patient cohorts, but which have not yet been applied in the field of MSI.

**Materials and Methods:**

In this study, the potential of integrative clustering is investigated for multilevel molecular MSI data to subdivide cancer patients into different prognostic groups. Metabolomic and peptidomic data are obtained by MALDI‐MSI from a tissue microarray containing material of 46 esophageal cancer patients. The integrative clustering methods Similarity Network Fusion, iCluster, and moCluster are applied and compared to non‐integrated clustering.

**Conclusion:**

The results show that the combination of multilevel molecular data increases the capability of integrative algorithms to detect patient subgroups with different clinical outcome, compared to the single level or concatenated data. This underlines the potential of multilevel molecular data from the same subject using MSI for subsequent integrative clustering.

Clustering algorithms are powerful data analysis methods to reveal the inherent relation of objects in a high‐dimensional feature space. Large, high‐dimensional datasets are now characteristic of most biomedical research owing to the widespread use of next‐generation sequencing and high‐throughput mass spectrometry. With such technologies, it is now possible to obtain high‐dimensional molecular information from many samples and many molecular levels (genome, epigenome, transcriptome, proteome, metabolome, etc.). In mass spectrometry imaging (MSI) for instance, matrix‐assisted laser desorption/ionization (MALDI) is able to detect, depending on the sample preparation procedure, metabolites, lipids, peptides, and proteins directly from tissues.^1^

The benefit of combining multilevel molecular data has been proven by the many studies of *The Cancer Genome Atlas*, where a comprehensive molecular description of cancer on different molecular levels (mostly genome, epigenome, transcriptome) has led to the discovery of new subgroups of patients with different molecular characteristics and different clinical outcomes.^2^

In all these examples, special clustering techniques have been employed that enable the integration of cross‐platform datasets, amongst them iCluster,^3^ intNMF,^4^ Similarity Network Fusion (SNF),^5^ and moCluster.^6^ There are many integrative clustering methods differing in their compatibility to certain data types, speed, and in their results if applied to the same data.^7^

In this study, we investigate the additive value of several integrative clustering methods on clustering multilevel molecular data obtained by MALDI‐MSI in order to find novel prognostic patient subgroups. This was done on high‐mass resolution MSI data from 46 primary resected esophageal tumors on a peptide and metabolite level, which were provided as a formalin‐fixed paraffin‐embedded (FFPE) tissue microarray (TMA). The TMA contains on average six tissue cores per patient (core size 0.6 mm). The patients gave their informed consent at the time of surgery and the local ethical commission of the Faculty of Medicine of the Technische Universität München, Germany, approved the use of the archival tissue for molecular analysis (No. 2136/08). The patients’ overall survival (median = 28 months) was calculated as the date of surgical resection to the date of death or last follow‐up.

FFPE tissue sample preparation for both metabolite and peptide MSI experiments has been done as reported previously^8^ and is described in detail in Supporting Information 1. Both MSI datasets were acquired at high‐mass resolution on Bruker Solarix FT‐ICR mass spectrometers (Bruker Daltonics, Billerica, USA) in MALDI mode. Metabolites were detected in negative ion mode in the mass range *m*/*z* 50–1000 and at a spatial resolution of 60 μm. Peptide measurements were performed in positive mode, in the *m*/*z* range 500–3000 at a spatial resolution of 70 μm. After measurements and matrix removal, the slides were stained with hematoxylin and eosin, scanned by a high‐resolution slide scanner (Mirax Desk, Zeiss, Germany) and coregistered to the MSI data, which allowed histology‐guided annotations of tumor areas. Samples were excluded from data analysis if they contained no tumor or if the tissue core section was lost during H&E staining. Together with the annotations, each dataset was uploaded separately to SCiLS Lab 2016b (Bruker Daltonics, Bremen, Germany). The further data pre‐processing is described in detail in Supporting Information 1, which included normalization, region merging, spectra processing, and peak picking. Finally, peak intensities for each patient's tumor were imported into the R statistical environment (v.3.4.2),^9^ where the data underwent standardization and filtering of sparse features and matrix‐ or trypsin‐related peaks. This resulted in 1801 peptide signals and 1764 metabolite‐related signals for investigating the additive value of integrative clustering for cancer patient subtyping (data available in Supporting Information).

Wang and Gu distinguish three integrative clustering strategies.^10^ Although from the group of *direct integrative clustering*, iCluster is very popular in cancer subtyping,^2^, ^11^ we also chose moCluster (R package “mogsa”) because it claims better performance.^6^ Both methods assume that the underlying biological structure in the data can be modeled by a reduced set of latent variables. In contrast, *clustering‐of‐clusters* methods first identify structures within each individual data type and then combine these structures. From this category, we chose SNF (R package “SNFtool”), also due to its application in cancer subtyping.^5^ It was not possible to use any method from the third category *regulatory integrative clustering* since it requires the prior knowledge on the identity of the features. Given the inclusion of 46 patients in the study, we limited the search for the optimum number of clusters to no more than five. The clustering was evaluated according to the differences in overall survival between the resulting patient groups. This was done by the log‐rank test (R package “survival”) with *p*‐values ≤ 0.05 being considered statistically significant. Finally, the results from the integrative clustering were contrasted to the results of submitting the individual or concatenated datasets to the respective integrative clustering techniques, if supported by the method.

**Clinical Relevance**

Biomedical and especially molecular cancer research move toward a more comprehensive molecular description of diseases in order to get hold of their biological complexity. For instance, The *Cancer Genome Atlas* (TCGA) initiate—mainly focused on sequencing technologies—has pursued that approach extensively in order to gather as much molecular information as possible per patient. The aims are a better understanding of the biological mechanisms and the complexity of cancer, as well as the detection of novel molecular patient subgroups with molecular characteristics that can be exploited for diagnostics or therapy. In these TCGA studies, the latter aim is achieved through the unsupervised and integrative clustering of the multilevel molecular data.

In this study, we want to give evidence on the usefulness of multilevel molecular data obtained by mass spectrometry imaging and combined with advanced integrative clustering methods, to enhance the detection of novel patients groups with clinical relevance, in this case with different prognosis.

To do so, SNF clustering was run on each individual dataset alone, the concatenated data, as well as the SNF‐fused data (details on parameterization of all subsequent clustering methods in Supporting Information 1). SNF is a graph‐based clustering which first represents the structure within each molecular class as similarity graphs based on the pair‐wise distances between each patient, and then integrates the graphs iteratively. Like any unsupervised clustering algorithm, SNF requires the number of expected patient groups to be selected in advance. If this is unknown, as here, statistical methods can be used to determine the number of clusters that best describes the data (but which does not necessarily represent biologically relevant groups). SNF uses the eigengap statistic. While the number of optimal clusters varied with the type of data (three for the metabolite data, and two for the peptide, the concatenated, and the SNF‐fused data), the SNF‐fused data was the only one achieving an unsupervised separation of the patients into groups with a significant difference in prognosis (*p* = 0.025; Figure 1D). SNF's internal feature ranking was used to extract the set of the 10% most relevant features, which is shown as heat map in Figure 1E,F and which contained most of the individually discriminating features as determined by classical statistical testing (Table S1 and Figure S1, Supporting Information).

**Figure 1:**
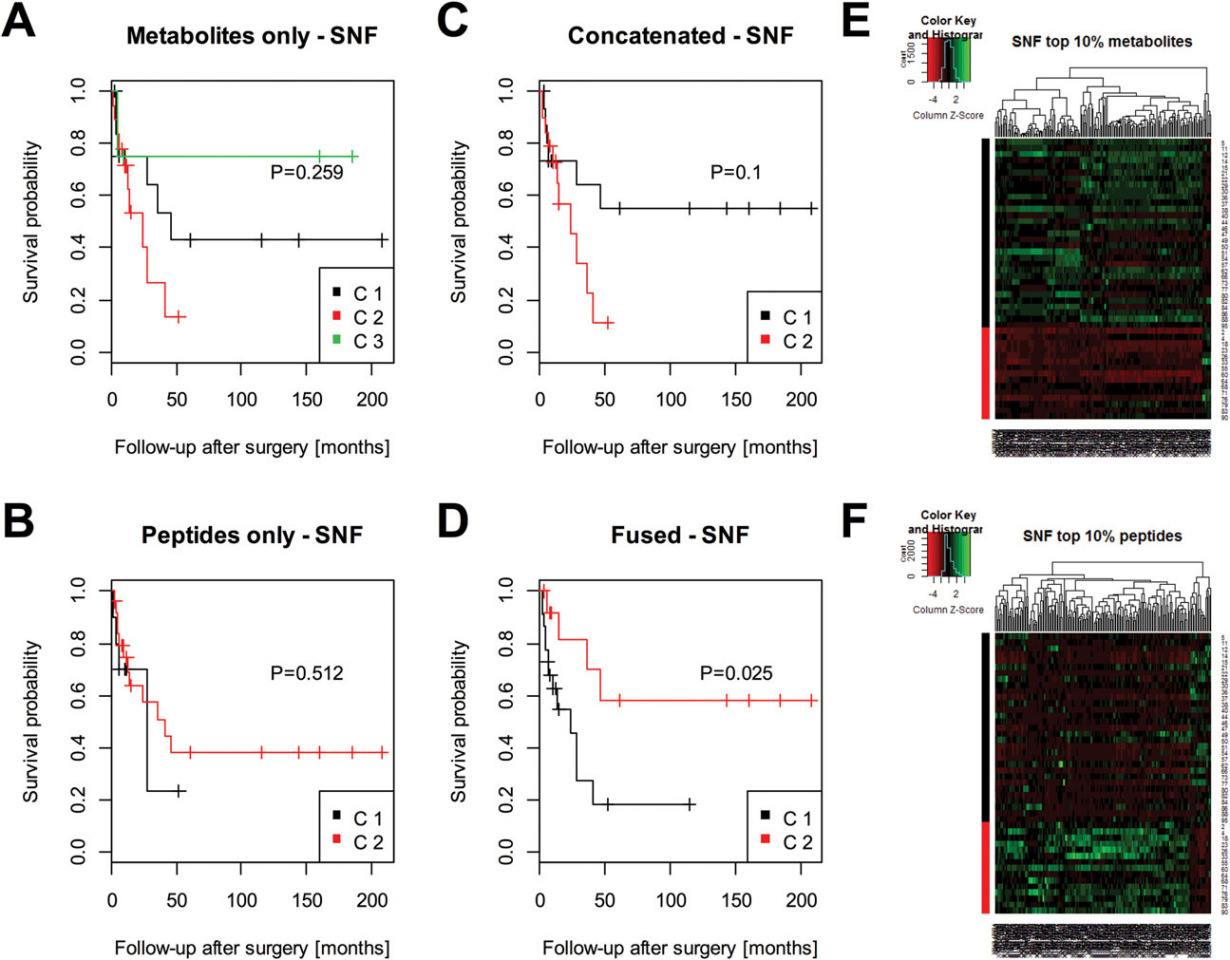
Integrative clustering by Similarity Network Fusion (SNF). Clustering by SNF was performed on the individual A) metabolite and B) peptide datasets. Differences in survival of the resulting patient groups were statistically evaluated by a Kaplan–Meier/log‐rank analysis. This was repeated for the C) concatenated metabolite/peptide data and D) the SNF‐fused data. Only the latter one resulted in the detection of patient groups with statistically significant differences in survival (*p* = 0.025). SNF also allows ranking features according to their relevance for the clustering. The top 10% E) most relevant metabolite and F) peptide signals are shown as heat maps where patients are in the rows and the features in the columns.

Next, moCluster was tested. It aims for the representation of the data in a lower‐dimensional space of latent variables by extending principle component analysis (PCA) to multiple data sets (consensus PCA), which can be further processed by ordinary clustering algorithms. Therefore, first the elbow method was used to select the minimum number of latent variables that explain most of the variance; in this case four (Figure 2A). The biggest positive change in the Gap‐statistic was used to specify the number of expected clusters, which gave five clusters (Figure 2B). Then the scores of the four latent variables were submitted to hierarchical clustering as supported by moCluster, and its dendrogram was cut into the expected number of clusters (Figure 2C). The five resulting groups, which are shown as a heat map in Figure 2E,F, differed significantly (*p* = 0.027) in their overall survival (Figure 2D). Similar results were not achievable with the metabolite data only (Figure S2, Supporting Information), the peptide data only (Figure S3, Supporting Information), or the concatenated data (Figure S4, Supporting Information).

**Figure 2:**
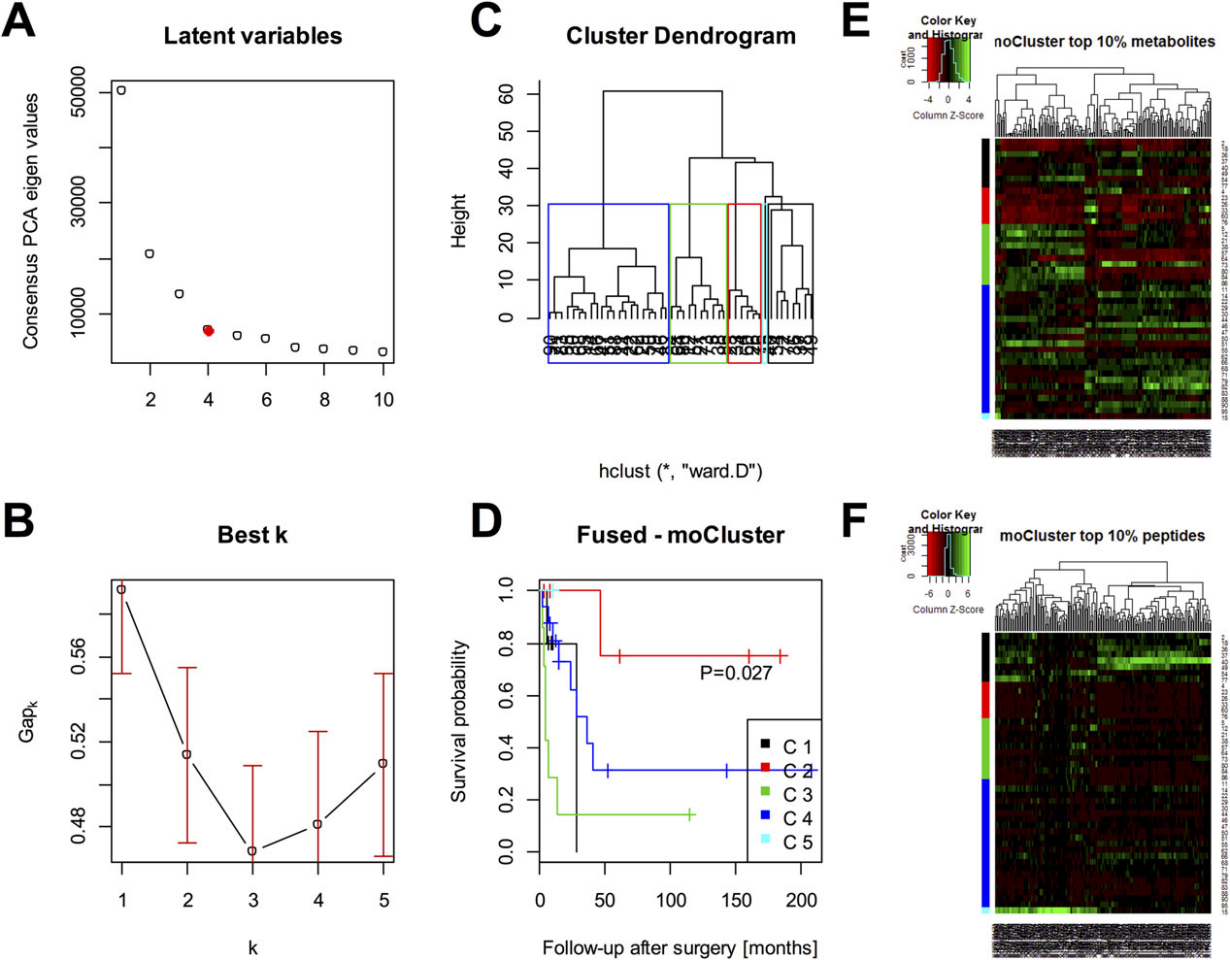
Integrative clustering by moCluster. A) First, four latent variables were found to represent sufficiently the concordant structures in the data based on their relative contribution of variance. The consensus‐PCA was then run with those four latent variables. B) The optimum number of clusters was found to be five where the Gap statistic shows the biggest positive change. C) The consensus‐PCA scores were submitted to hierarchical clustering and the resulting tree was cut at the height (≈30) where five patient groups are obtained. D) These patient groups differed statistically significant in their survival (*p* = 0.027) as calculated by Kaplan–Meier/log‐rank analysis. moCluster also provides a ranking of the features according to their loading. The top 10% E) most relevant metabolite and F) peptide signals are shown as heatmaps where patients are in the rows and the features in the columns.

Also iCluster was tested, but did not result in the detection of groups with different prognosis (Figure S5, Supporting Information). All results are summarized in Table 1. Interestingly, different integrative clustering techniques exhibited different resolving powers with respect to the optimum number of patient groups with different prognosis. SNF tended to identify differences in survival for a lower number of clusters (Figure 1D), whereas moCluster data tended to identify more than two prognostic groups (Figure 2D).

**Table 1:** Clustering and survival analysis results

Clustering method	Dataset	Optimal number of clusters	Log rank *p*‐value
SNF	Metabolites	3	0.259
	Peptides	2	0.512
	Concatenated	2	0.100
	Fused	2	0.025^a)^
iCluster	Fused	2	0.134
moCluster	Metabolites	5	0.152
	Peptides	5	0.249
	Concatenated	4	0.100
	Fused	5	0.027*

a Considered statistically significant (*p* ≤ 0.05)

Once patient groups with different clinical outcome have been identified, it is of interest to get an insight into the predominating biological processes and molecular actors within each group. Disposing of high‐mass accuracy mass spectrometry data, we obtained tentative assignments for the observed *m*/*z* species by mass matching them with a tolerance of 5 ppm against the Human Metabolome Database and the Matisse tissue proteome database (Supporting Information 1).^12^ This resulted in tentative assignments of 46.8% of the peptides and 2.5% of the metabolites (Table S2, Supporting Information). Filtering according to the 10% most relevant features of the SNF clustering, a reduced list of metabolites (*n* = 13) and peptides (*n* = 128) was submitted, together with their average intensities per class, to Reactome.org for pathway analysis. Most SNF clustering‐relevant pathways were found related to oncogenic MAPK, BRAF, and RAF signaling, innate immune system, and apoptosis (false discovery rate ≤ 0.01, Supporting Information 3).

Besides the biological interpretation of the data, it is also of interest to investigate the ability of the data to assign a new patient to one of the prognostic groups without performing the clustering again. SNF offers an internal routine for supervised classification. Using this routine and a leave‐one‐patient‐out cross‐validation for testing, an accuracy of 91.3% was achieved, thereby indicating the robustness of the found clusters for accurate prediction of the clinical outcome of a patient (Supporting Information 1).

To conclude, MALDI‐MSI continues its development toward higher spatial and higher mass resolution, and the ability to analyze even more molecular classes. Currently MALDI‐MSI can be used to analyze acidic metabolites, lipids, glycans, endogenous peptides, proteolytic peptides, and proteins. To date the complementarity between these different molecular classes, for example for improved differentiation between patient subgroups and understanding of the origin of the differences, has not been adequately utilized. Here it was demonstrated, for the first time in MSI, that integrative clustering increased the ability of MSI to differentiate patient subgroups beyond that achievable using the metabolite/peptide datasets in isolation. Such approaches have great potential to extract the most out of this new multilevel molecular type of data.

## Conflict of Interest

The authors declare no conflict of interest.

## Supporting information

Supporting Data

Supporting Information

Supporting Information

Supporting Information

Supporting Figure S1

Supporting Figure S2

Supporting Figure S3

Supporting Figure S4

Supporting Figure S5

Supporting Table S1

Supporting Table S2
