# Supplementary material for: Integrative Clustering in Mass Spectrometry Imaging for Enhanced Patient Stratification
Source: Proteomics Clin Appl. 2019 Jan 4;13(1):1800137. doi: 10.1002/prca.201800137 (PMC6590511; doi:10.1002/prca.201800137)
Supplement: Supplementary file 2 — Supporting Information [file PRCA-13-na-s002.docx]

SUPPORTING INFORMATION 1:

# Integrative Clustering in Mass Spectrometry Imaging for Enhanced Patient Stratification

Benjamin Balluff^1^, Achim Buck^2^, Marta Martin-Lorenzo^1^, Frédéric Dewez^1^, Rupert Langer^3^, Liam A. McDonnell^4^, Axel Walch^2^, Ron M.A. Heeren^1^

**Corresponding author:**

Benjamin Balluff, Maastricht University, The Maastricht MultiModal Molecular Imaging institute (M4I), Universiteitssingel 50, pigeon hole 57, P.O. Box 616, 6200 MD Maastricht, The Netherlands; Phone: +31 43 388 1251; Email: [b.balluff@maastrichtuniversity.nl](mailto:b.balluff@maastrichtuniversity.nl)

# MATERIAL AND METHODS

## Material

A formalin-fixed paraffin-embedded (FFPE) tissue microarray (TMA) of esophageal adenocarcinomas from 46 primary resected tumors was used for this study. The TMA contains on average six tissue cores per patient (core size 0.6 mm). Samples were collected between 1990 and 2011 from patients who did not undergo neoadjuvant therapy. The resection specimens were processed in a highly standardized manner, opened immediately after surgery, fixed in 4% (vol/vol) neutral-buffered formalin and paraffin wax-embedded using automated procedures. The patients gave their informed consent at the time of surgery and the local ethical commission of the Faculty of Medicine of the Technische Universität München, Germany, approved the use of the archival tissue for molecular analysis (No. 2136/08). The patients’ overall survival (median=28 months) was calculated as the date of surgical resection to the date of death or last follow-up.

## Slide preparation

Two consecutive 6 µm sections were made on a microtome (HM325, Microm, Germany) and mounted onto 1:1 (v/v) poly-L-lysine coated:0.1% Nonidet P-40 pretreated (Sigma-Aldrich, Munich, Germany) indium-tin-oxide glass slides (Bruker Daltonics, Bremen, Germany). These sections were further adhered to the slide by warming on a heating block at 60°C for 1 hour.

## Metabolite experiments

FFPE tissue sample preparation for metabolite experiments was done as described previously [Ly A et al. “High-mass-resolution MALDI mass spectrometry imaging of metabolites from formalin-fixed paraffin-embedded tissue”, Nat Protoc. 2016 Aug;11(8):1428-43. doi: 10.1038/nprot.2016.081]. Briefly, paraffin was removed by two subsequent 8 min xylene washes followed by a drying at room temperature on a 40°C hot plate and the application of fiducial markers. The matrix, 10 mg/ml 9-aminoacridine hydrochloride monohydrate (Sigma-Aldrich, Munich, Germany) in 70% methanol, was applied onto the sample with the a SunCollect spraying system (SunChrom, Friedrichsdorf, Germany) using the following parameters: x=0.5 mm; y=2.0 mm; z=20 mm; speed(x,y)=med(1)=900 mm/min; flowrates: layers 1 to 3 at 10, 20, and 30 µL/min, respectively, and layers 4 to 8 at 40 µL/min. High-mass resolution MALDI-MSI was carried out on a Bruker Solarix 7T FT-ICR mass spectrometer (Bruker Daltonics), controlled by solariXcontrol (v.1.5.0, Bruker Daltonics) and ﬂexImaging (v.4.0, Bruker Daltonics). Data was acquired in negative ion mode in the mass range m/z 50–1000 with a 1M data point transient (0.367 s duration) and an estimated resolution of 49,000 at *m/z* 400. The spatial resolution was set to 60 μm. The Smartbeam-II Nd:YAG (355 nm) laser frequency was set to 1000 Hz and 100 shots were accumulated per spot. L-Arginine was used for external calibration.

## Peptide experiments

For tryptic peptide experiments, paraffin was removed by three xylene washes (5, 10, and 10 min). Then the slides were washed three times (each 2 min) in 100% ethanol and twice (each 5 min) in ultrapure MilliQ water. Antigen-retrieval was performed with 10mM citric acid monohydrate at pH 6 as buffer in the Antigen Retriever 2100 (Aptum Biologics, Southampton, United Kingdom) for 20 min. After antigen-retrieval, slides were rinsed twice for 1 min in deionized water and dried in a desiccator. Fiducial markers were placed on the slide. A 0.02 µg/µL trypsin solution was sprayed with the SunCollect spraying system (SunChrom) with the following parameters: z=25 mm; d=1.0mm; speed=900 mm/min and a constant flow rate of 10 µL/min during the 15 layers. Incubation of the slide was done for 17 hours at 37°C in a saturated environment using an airtight box filled with 100 mL of 50% MeOH and 50% MilliQ water. The matrix (5 mg/mL alpha-cyano-4-hydroxycinnamic acid in 50% acetonitrile/0.2% trifluoroacetic acid) was applied with the SunCollect sprayer (SunChrom) using the following parameters: z=25 mm; d=2.0mm; speed=1390 mm/min; and flowrates: layers 1 to 3 at 10, 20, and 30 µL/min, respectively, and layers 4 to 7 at 40 µL/min.

Peptide measurements were performed in positive mode, in the *m/z* range 500-3000 with a Bruker Solarix 9.4T FT-ICR mass spectrometer (Bruker Daltonics) at a spatial resolution of 70 µm.

## Histological annotation

After peptide or metabolite measurements, the matrix was removed from the slides with 70% ethanol and stained with hematoxylin and eosin (H&E). Optical images from the slides were obtained by a high-resolution slide scanner (Mirax Desk, Zeiss, Germany) and coregistered to the MSI data in the flexImaging software (Bruker Daltonics) using the fiducial markers. Histology-guided tumor annotation was done by defining regions-of-interest in flexImaging. Samples were excluded from data analysis if they contained no tumor or if the tissue core section was lost during H&E staining. Together with the annotations, each dataset was uploaded separately to SCiLS Lab (v. 2016b, Bruker Daltonics).

## Data pre-processing metabolites

Average spectra of the defined tumor regions were normalized to the root mean square (RMS) value and exported from flexImaging to MATLAB 2014b (MathWorks, Natick, MA, USA) for peak detection as described previously [Ly A et al. “High-mass-resolution MALDI mass spectrometry imaging of metabolites from formalin-fixed paraffin-embedded tissue”, Nat Protoc. 2016 Aug;11(8):1428-43. doi: 10.1038/nprot.2016.081]. All exported spectra underwent baseline subtraction, resampling to lower data dimensionality, and smoothing to remove tissue and measurement artefacts. Peak picking was conducted using a modiﬁed version of the LIMPIC algorithm with a minimal peak width of m/z 0.0005, an S/N of 2 and an intensity threshold of 0.01% compared to the base peak. The peak list was finally imported into SCiLS Lab and RMS-normalized peak intensities for each patient’s tumor regions were exported as CSV file.

## Data pre-processing peptides

The peptide data was first normalized to the RMS in SCiLS Lab (2016b). Then joining all individual tumor regions created a tumor average spectrum across all patients. This spectrum was exported to MATLAB 2017b (MathWorks, Natick, MA, USA) for undergoing the following processing:

- First, the spectrum underwent high-pass filtering using top-hat filtering with a window size of 5 to remove artifact signals:


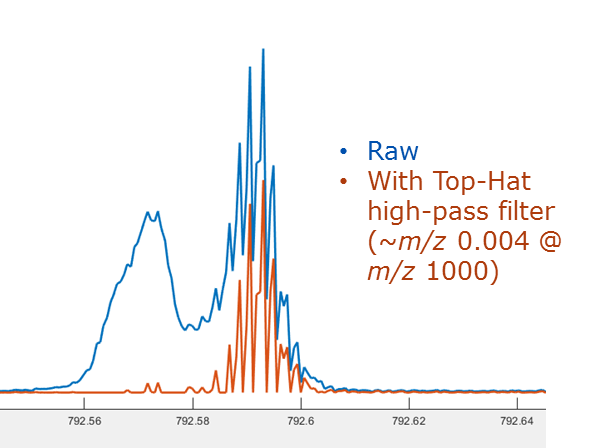


- Peak picking was done with *mspeaks* (Bioinformatics toolbox) with a minimum relative intensity to the base peak of 0.2%. Peak locations were determined at 90% height:

absolute_int_threshold = max(intensities)*relative_int_threshold;

[Peaklist, PFWHH, PExt] = mspeaks(mz, intensities, 'HeightFilter', absolute_int_threshold, 'PeakLocation', 0.9, 'Denoising', false);

- Apodization peaks were removed by searching defined *m/z* [3 ppm to 25 ppm] and intensity windows [0% to 10% peak intensity] on the right and left side of each peak:


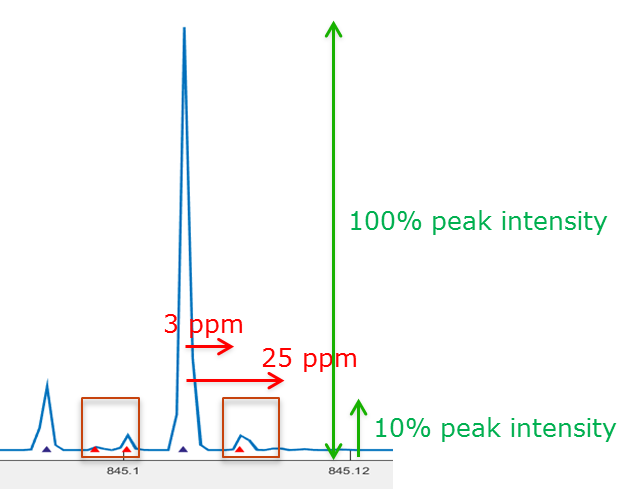


- Isotope signals were detected using the averagine approach. The averagine is an estimation of the average amino acid. Based on the observed mass, the number of averagines can be calculated and therefore also an estimation of the elemental composition of the peptide. This allows further computing a theoretical isotopic distribution for each observed *m/z* value. Every peak is then investigated for subsequent peaks that match the estimated isotopic distribution with certain tolerances: the difference in intensity between observed and expected isotope can be maximum 70% and the difference in observed and expected *m/z* value can be maximum *m/z* 0.02. Signals identified as isotopic signals were removed:


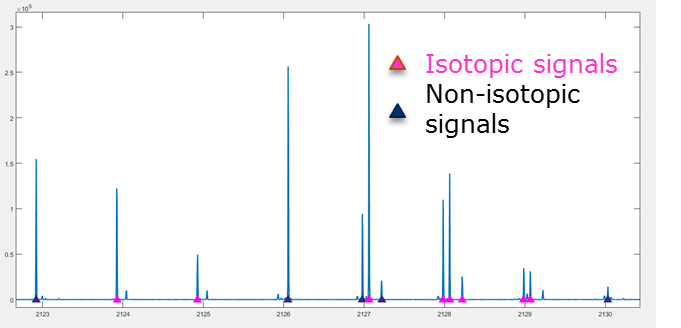


- Finally, matrix related signals were also removed. These were identified by a correlation analysis to the matrix peak at where peaks with a Pearson correlation coefficient >= 0.5 were considered matrix-related:


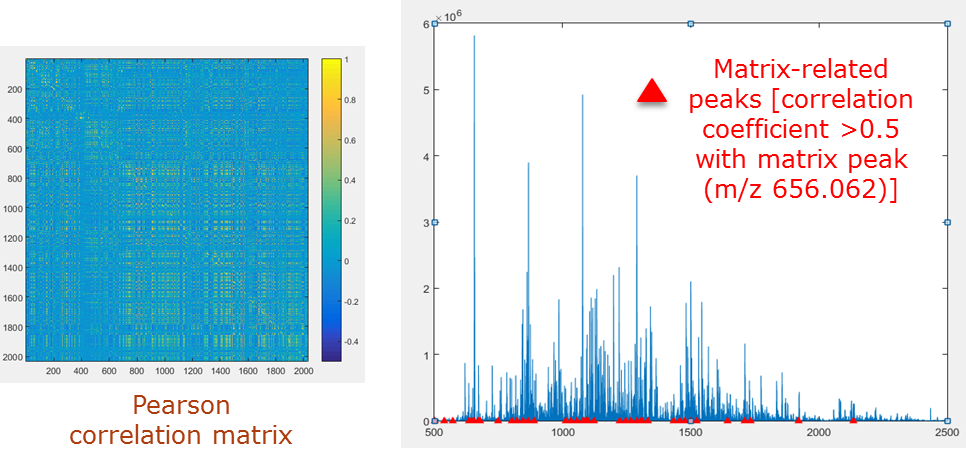


This adjusted peak list was reimported into SCiLS Lab and RMS-normalized peak intensities for each patient’s tumor regions were exported as CSV file and imported into the R statistical environment (v. 3.4.2).

## Integrative clustering

In R, matrix (*m/z* 193.0775) related peaks (Pearson correlation coefficient > 0.5) were removed from the metabolite data, as well as zero-rich features (≥30%) and trypsin (*m/z* 842.5097) related peaks (Pearson correlation coefficient > 0.8) from the peptide data. All remaining features were z-scored before analysis. If not mentioned otherwise, standard parameterization was used for all subsequently described methods. The R code is provided in the Supporting Information 2.

Similarity Network Fusion [SNF] (R package “SNFtool”), iCluster (R package “iCluster”), and moCluster (R package “mogsa”) were used for integrative clustering. SNF was run with the parameters: number of neighbors=10, alpha=0.5, and number of iterations=15. After fusion, the optimal number of clusters was estimated by SNF with the eigen-Gap statistic.

iCluster was run with the parameterization: lambda=0, 0.01, and 0.2 and a maximum number of iterations of 3. The optimal number of clusters was determined using the method internal proportion of deviance (POD) statistic.

In contrast to SNF or iCluster, moCluster is a multi-step process. Briefly, first an initial consensus PCA was done with ten components followed by a permutation test to identify the number of latent variables that are necessary to represent the concordant structures sufficiently in the datasets. Four components were chosen with 20% non-zero coefficients for the variable loading vectors. Hierarchical clustering was done on the resulting consensus PCA scores with Euclidean distance and Ward’s linkage. The optimal number of data clusters was estimated at the maximum positive change in the Gap statistic.

Given the inclusion of 46 patients in the study, the search for the optimum number of clusters was limited to not more than five clusters for every method.

The resulting clusters, i.e. subgroups of patients, were evaluated according to their differences in overall survival between the patients. This was done by the log rank test (R package “survival”) with P-values ≤ 0.05 being considered statistically significant.

Finally, the results from the integrative clustering were contrasted to the results of submitting the individual or concatenated datasets to the respective integrative clustering techniques, if supported by the method (Table 1).

## Classification and discriminatory power of features

Some integrative clustering methods allow assessing the individual contribution of each feature to the clustering result by internal feature-ranking routines. SNF uses the normalized mutual information (NMI) and moCluster the loading score in the latent variable space to quantify the relevance of each feature. We contrasted those internal rankings with the discriminatory power of every single feature as determined by an ANOVA with subsequent multiple testing correction using Holm’s method (Figure S1, Supporting Information).

Once clusters with different clinical outcome have been identified it is of interest to define supervised classifiers in order to be able to assign new unknown patients to the prognostic groups without having to perform the clustering again. We have done this for the SNF clustering results using SNF’s internal class prediction routine ‘groupPredict’ and a leave-one-patient-out cross-validation to evaluate the classification accuracy, sensitivity (defining the poor prognosis group as ‘positive’ class), and specificity (Supporting Information 2).

## Tentative identifications and pathway analysis

Tentative identifications of the observed metabolite and peptide signals were obtained by querying the single observed *m/z* values via mass matching against theoretical masses of metabolites in the Human Metabolome Database (HMDB; version 4; October 2018; http://www.hmdb.ca) and peptides in the Matisse databases. We considered only endogenous metabolites in the HMDB (n=16,990), and only peptides detected in esophageal carcinoma in Matisse (n=9,155), which is a tissue specific database for bottom-up and top-down mass spectrometry imaging experiments [Maier S et al. “Comprehensive identification of proteins from MALDI imaging”, Mol Cell Proteomics. 2013 Oct;12(10):2901-10. doi: 10.1074/mcp.M113.027599]. The latter uses the discontinued IPI (international protein index) as protein identifier. We therefore mapped the IPI id to the UniProt id using the cross-references file for human IPI ids (release 3.87).

Mass matching based on high-mass accuracy data is a common approach in the field of mass spectrometry imaging for obtaining tentative identifications of peptides, lipids, and metabolites [Heijs B et al. “Comprehensive analysis of the mouse brain proteome sampled in mass spectrometry imaging”, Anal Chem. 2015 Feb 3;87(3):1867-75. doi: 10.1021/ac503952q; Ly A et al. “High-mass-resolution MALDI mass spectrometry imaging of metabolites from formalin-fixed paraffin-embedded tissue”, Nat Protoc. 2016 Aug;11(8):1428-43. doi: 10.1038/nprot.2016.081]. Since the local estimated mass errors were below 1 ppm for the peptides at ~ 1000 Da (observed: *m/z* 842.5097; theoretical trypsin auto-cleavage product: *m/z* 842.5094) and below 2 ppm for the metabolites at ~ 200 Da (observed: *m/z* 193.0775; theoretical 9-aminoacridine: m/z 193.0771), we assumed a mass error of maximum 5 ppm for the entire mass range of both molecular classes.

We therefore compared our experimental *m/z* values within a maximum tolerance of 5 ppm to the theoretical *m/z* values in the databases assuming most the common ionization products for the metabolite experiments ([M-H]^-^, [M+Na-2H]^-^, and [M+K-2H]^-^) and for the peptide experiments ([M+H]^+^, [M+Na]^+^, and [M+K]^+^).

Tentatively identified metabolites and peptides, which were also within the 10% most relevant features according to the SNF internal feature ranking, were then submitted together with their average intensities per cluster to Reactome.org (release 66, 2018-11-21) for identifying pathways that are related to the clustering (Supporting Information 3).
