# Supplementary material for: Integrative Clustering in Mass Spectrometry Imaging for Enhanced Patient Stratification
Source: Proteomics Clin Appl. 2019 Jan 4;13(1):1800137. doi: 10.1002/prca.201800137 (PMC6590511; doi:10.1002/prca.201800137)
Supplement: Supplementary file 4 — Supporting Information [file PRCA-13-na-s004.pdf]

reactome

Pathway Analysis Report

Tentative.id

This report contains the pathway analysis results for the submitted sample 'Tentative.id'. Analysis was performed against Reactome version 66 on 21/11/2018 using any resource identifiers for the mapping. The web link to these results is:

https://reactome.org/PathwayBrowser/#/ANALYSIS=MjAxODExMjAxMzI3NDNfNzE1OQ%3D%3D

Please keep in mind that analysis results are temporarily stored on our server. The storage period depends on usage of the service but is at least 7 days. As a result, please note that this URL is only valid for a limited time period and it might have expired.

Table of Contents

1. Introduction
2. Properties
3. Genome-wide overview
4. Most significant pathways
5. Pathway details
6. Identifiers found
7. Identifiers not found

1. Introduction

Reactome is a curated database of pathways and reactions in human biology. Reactions can be considered as pathway 'steps'. Reactome defines a 'reaction' as any event in biology that changes the state of a biological molecule. Binding, activation, translocation, degradation and classical biochemical events involving a catalyst are all reactions. Information in the database is authored by expert biologists, entered and maintained by Reactome's team of curators and editorial staff. Reactome content frequently cross-references other resources e.g. NCBI, Ensembl, UniProt, KEGG (Gene and Compound), ChEBI, PubMed and GO. Orthologous reactions inferred from annotation for Homo sapiens are available for 17 non-human species including mouse, rat, chicken, puffer fish, worm, fly, yeast, rice, and Arabidopsis. Pathways are represented by simple diagrams following an SBGN-like format.

Reactome's annotated data describe reactions possible if all annotated proteins and small molecules were present and active simultaneously in a cell. By overlaying an experimental dataset on these annotations, a user can perform a pathway over-representation analysis. By overlaying quantitative expression data or time series, a user can visualize the extent of change in affected pathways and its progression. A binomial test is used to calculate the probability shown for each result, and the p-values are corrected for the multiple testing (Benjamini–Hochberg procedure) that arises from evaluating the submitted list of identifiers against every pathway.

To learn more about our Pathway Analysis, please have a look at our relevant publications:

Fabregat A, Sidiropoulos K, Garapati P, Gillespie M, Hausmann K, Haw R, … D'Eustachio P (2016). The reactome pathway knowledgebase. Nucleic Acids Research, 44(D1), D481–D487. https://doi.org/10.1093/nar/gkv1351.

Fabregat A, Sidiropoulos K, Viteri G, Forner O, Marin-Garcia P, Arnau V, … Hermjakob H (2017). Reactome pathway analysis: a high-performance in-memory approach. BMC Bioinformatics, 18.

2. Properties

- This is an **expression** analysis: The numbers are used to produce a scaled coloured overlay over Reactome pathway diagrams, as a means to visualize relative expression levels. Note that the numeric values do not have to be expression data, for instance by using gene association scores the same analysis can be used to visualize genotyping results.
- 106 out of 141 identifiers in the sample were found in Reactome, where 595 pathways were hit by at least one of them.
- All non-human identifiers have been converted to their human equivalent.
- This report is filtered to show only results and pathway diagrams for Homo sapiens.
- The unique ID for this analysis (token) is MjAxODExMjAxMzI3NDNfNzE1OQ%3D%3D. This ID is valid for at least 7 days in Reactome's server. Use it to access Reactome services with your data.
