# Supplementary figures and images for: Integrative Clustering in Mass Spectrometry Imaging for Enhanced Patient Stratification
Source: Proteomics Clin Appl. 2019 Jan 4;13(1):1800137. doi: 10.1002/prca.201800137 (PMC6590511; doi:10.1002/prca.201800137)

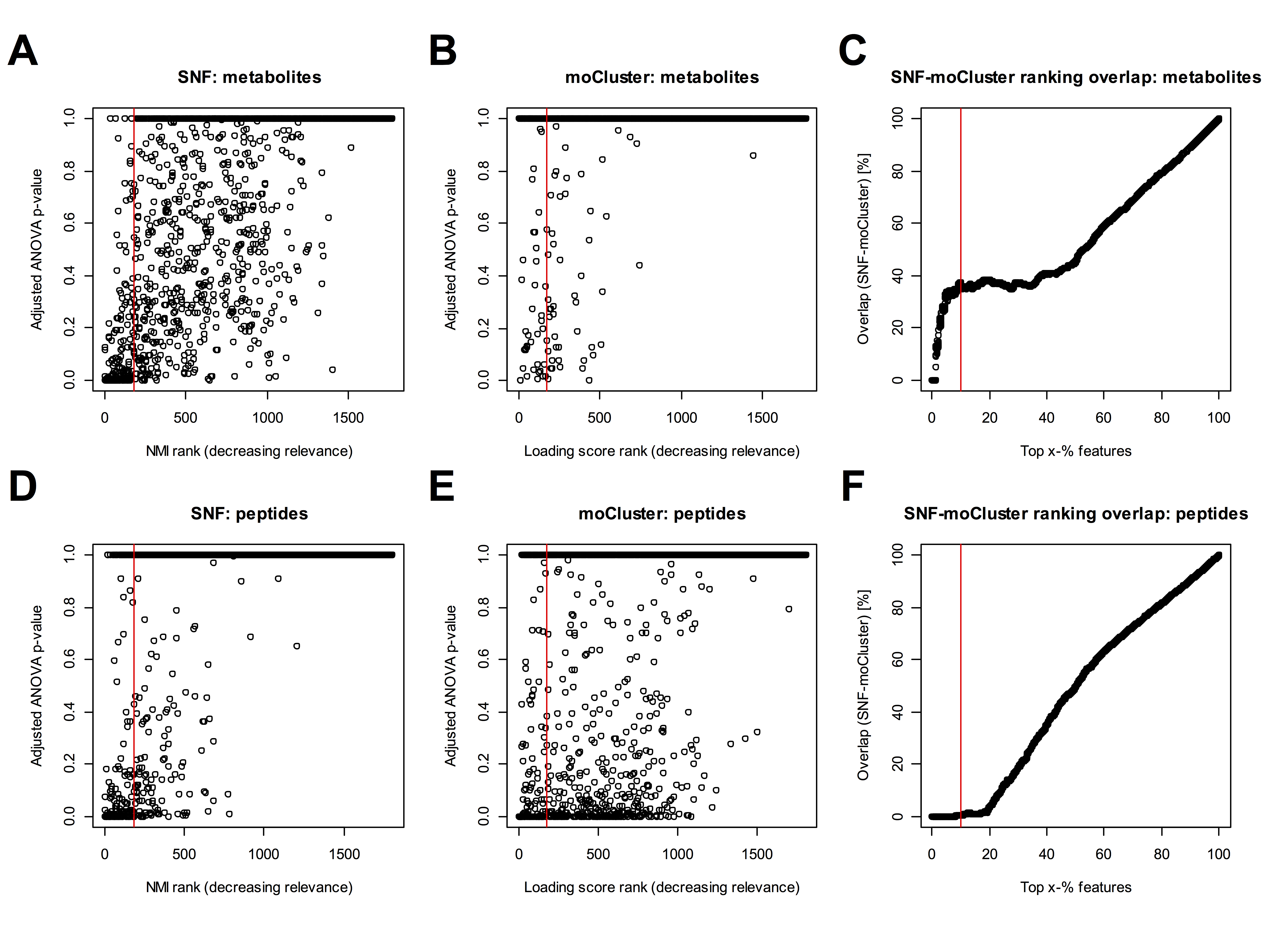

Supplement: Supplementary file 5 — Supporting Figure S1 [file PRCA-13-na-s005.tiff]

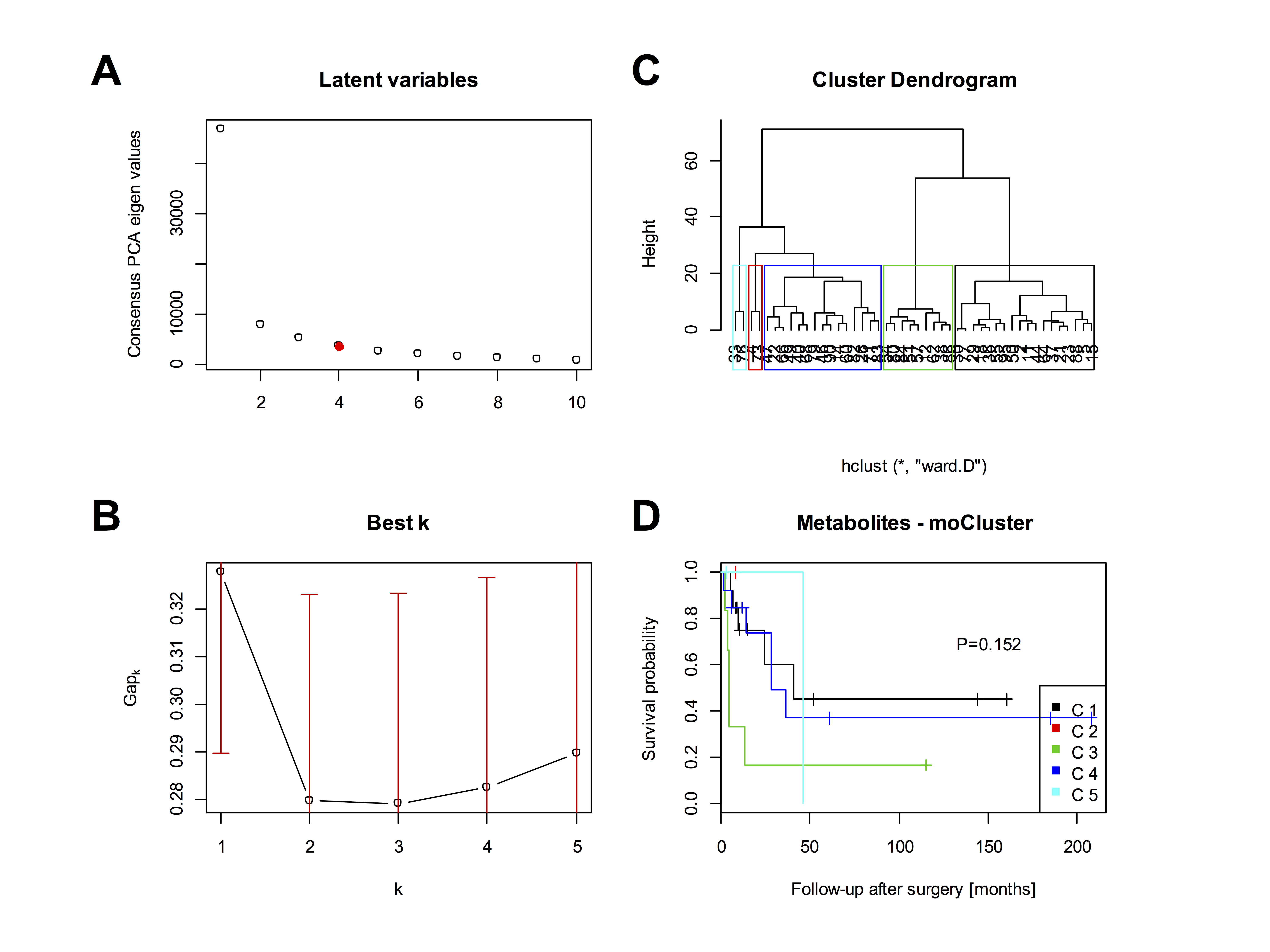

Supplement: Supplementary file 6 — Supporting Figure S2 [file PRCA-13-na-s006.tiff]

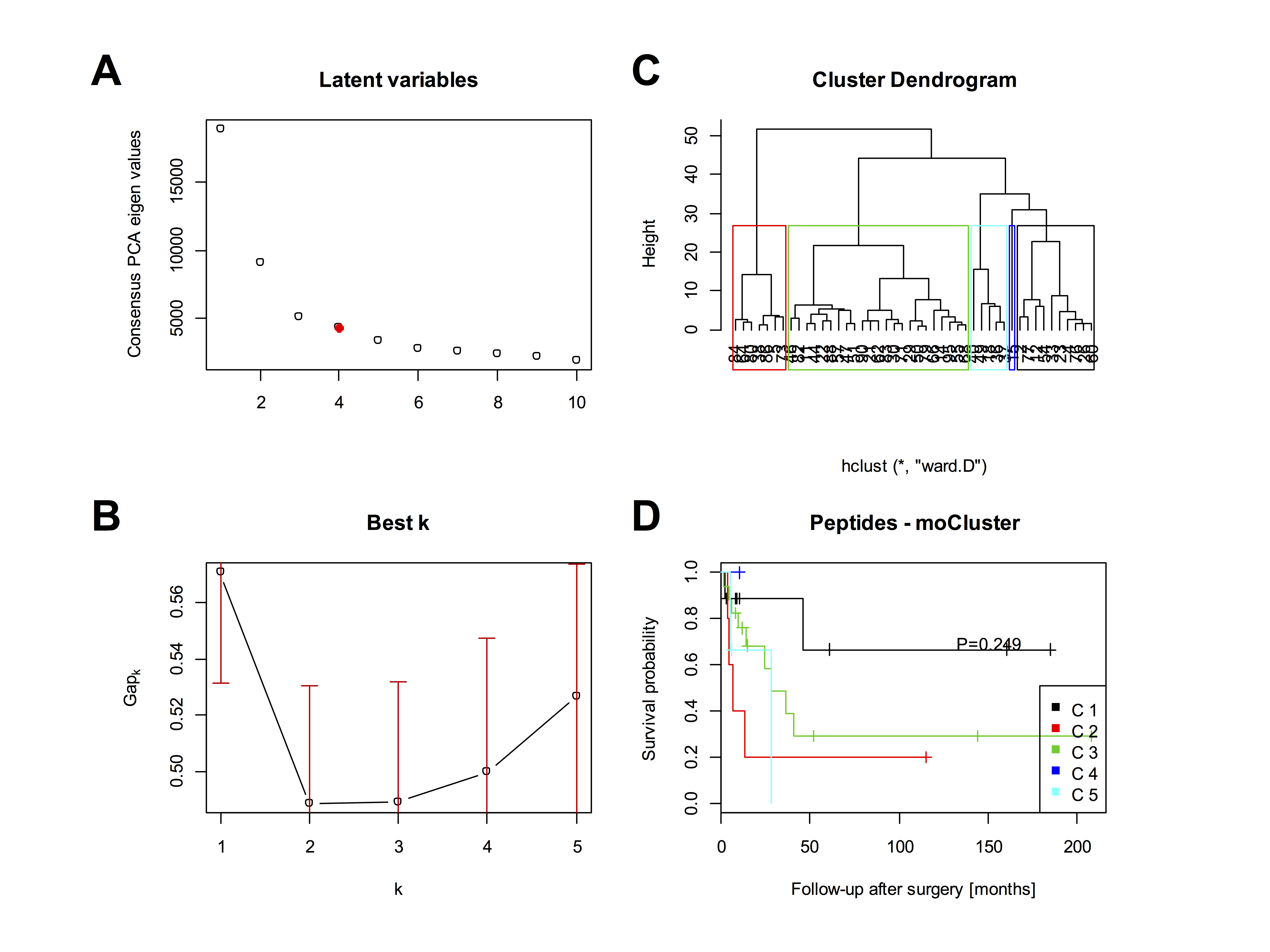

Supplement: Supplementary file 7 — Supporting Figure S3 [file PRCA-13-na-s007.tiff]

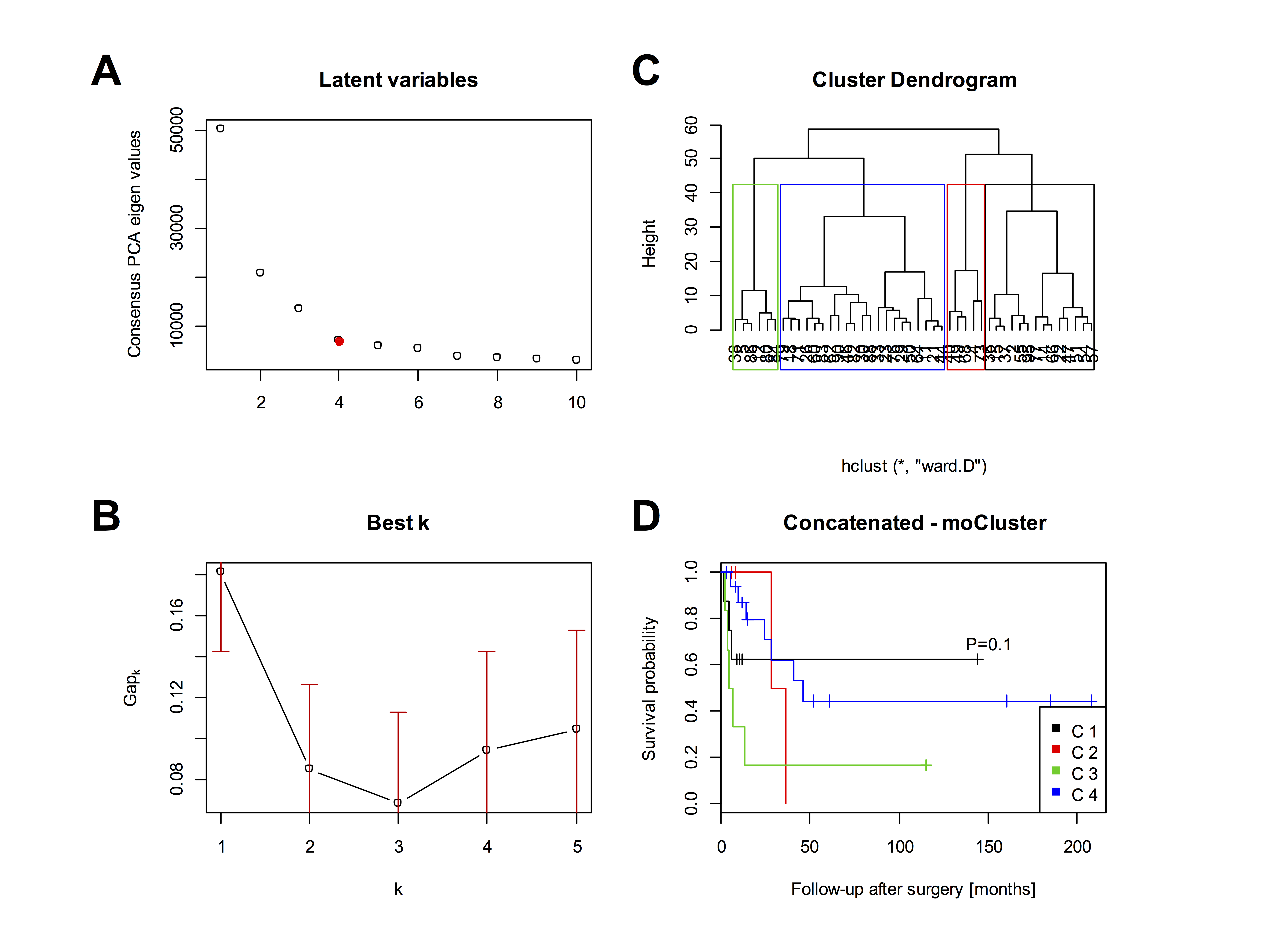

Supplement: Supplementary file 8 — Supporting Figure S4 [file PRCA-13-na-s008.tiff]

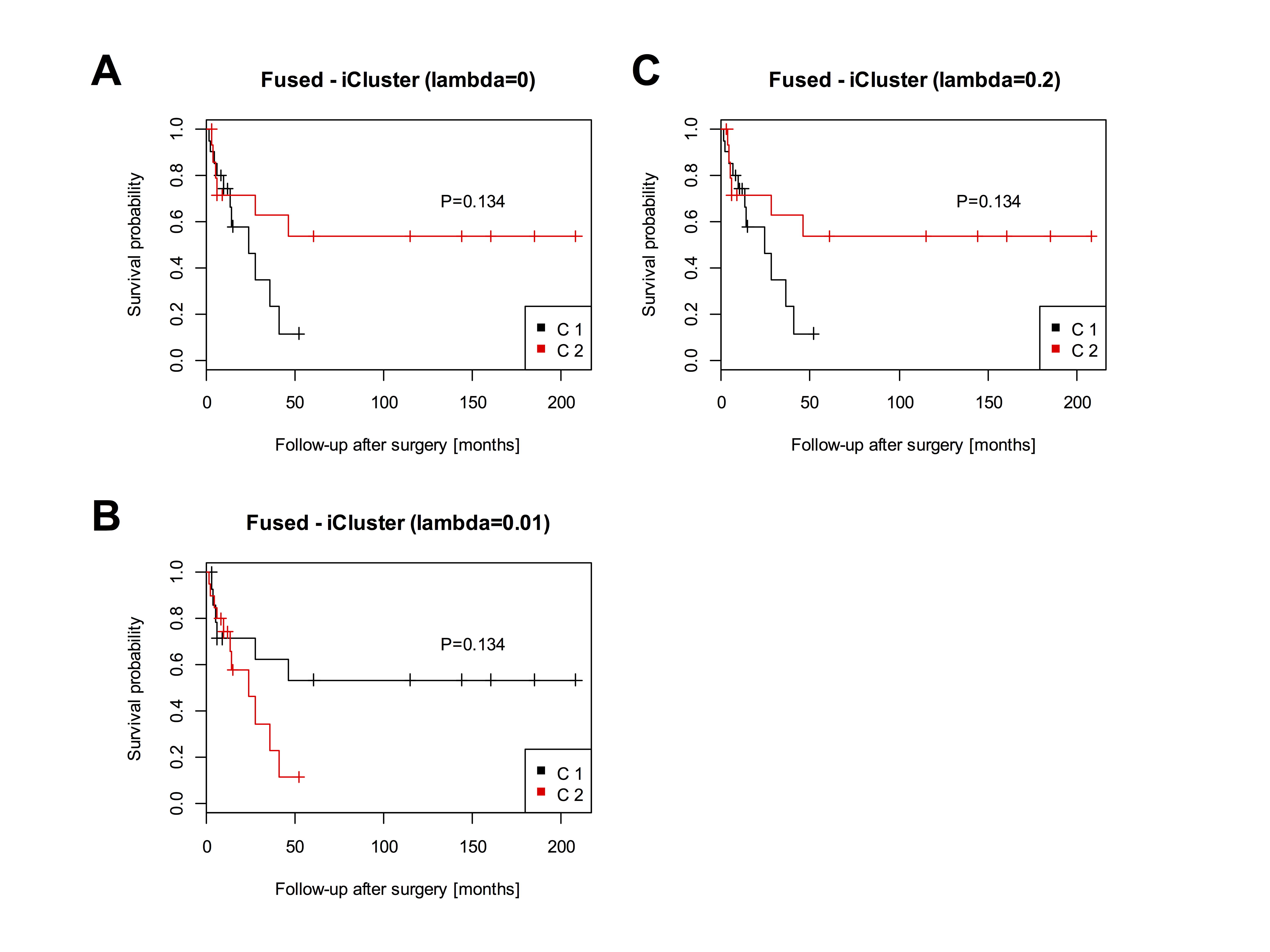

Supplement: Supplementary file 9 — Supporting Figure S5 [file PRCA-13-na-s009.tiff]
